# Indigenous Knowledge, Processing Method, and Future Potential of *Areke* and *Tella*: Traditional Fermented Beverages in Ethiopia

**DOI:** 10.1155/ijm/1765475

**Published:** 2026-06-19

**Authors:** Mulugeta Fentahun Nega

**Affiliations:** ^1^ Department of Biology, College of Natural and Computational Sciences, Debre Markos University, Debre Markos, Amhara, Ethiopia, dmu.edu.et

**Keywords:** *Areke*, Ethiopia, processing method, *Tella*, traditional fermented beverages

## Abstract

In Ethiopia, there are many forms of art in the area of local alcoholic fermentation. Beverages made locally using indigenous knowledge are known as traditional fermented alcoholic beverages. Local fermented alcoholic beverages are consumed by diverse ethnic groups in the country. Different traditional beverages made with fermentation in Ethiopia, such as *Shameta*, *Borde*, *Imbushbush*, *Winetej*, *Tella*, *Tej*, *Keribo*, *Korefe*, *Buqri*, *Duka,* and distilled spirits such as *Katikala* or *Areke*. Traditional *Tella* and *Areke* alcoholic beverages are the most popular homemade beverages, made on a small scale and sold by local alcohol vendors from their houses. The process of fermentation is uncontrolled, spontaneous, and natural. The most common microorganisms found during the fermentation of these indigenous alcoholic beverages are lactic acid bacteria and yeasts. *Areke* is a locally produced distilled alcoholic beverage produced from the results of fermentation that are made almost identically to *Tella* with the exception of the fermentation mass. The three fundamental steps in the *Tella* preparation procedure such as “tenses,” “difdif,” and “Tella.” The growing interest in traditional and functional beverages worldwide promises upgrading and commercialization of *Areke* and *Tella* by standardizing the processes, which could create local employment, generate income, and promote cultural tourism. However, challenges such as a lack of standardization, inconsistent quality, and insufficient scientific evidence must be addressed. To transition these traditional beverages into industrial production, further research is required. This review summarizes scientific information on indigenous knowledge, processing methods, microbial dynamics, metabolic profiling, functional properties, and the future commercial potential of *Areke* and *Tella*, traditional fermented beverages in Ethiopia.

## 1. Introduction

The origin of the first fermented beverage remains unknown. However, it was probably discovered by ancient communities accidentally [1]. Native communities in various countries worldwide drink a variety of indigenous alcoholic beverages made locally. These kinds of beverages are widely manufactured as homemade beverages in numerous African nations in a number of methods [2]. Local beverage production is related to the enormous indigenous knowledge in the region, with the knowledge extricable linked to its institutional, social, cultural, and environmental contexts [3]. Traditional beverage processing practices are an essential part of the indigenous knowledge that has been passed down through generations from parent to child. Unfortunately, this vital body of indigenous knowledge is often undervalued [4].

Ethiopia produces and consumes enormous amounts of traditional fermented beverages. *Buqri*, *Shameta*, *Borde*, *Imbushbush*, *Winetej*, *Keribo*, *Korefe*, *Tella*, *Tej*, *Duka*, and distilled spirits such as *Areke or Katikala* are among the many traditional fermented beverages that Ethiopians consume [5–7]. *Tella*, *Borde*, local *Areke* (*Katikala*), *Shamita*, *Cheka* and *Korefe* are among Ethiopia′s most well‐known homemade fermented alcoholic beverages. The most popular beverages in central Ethiopia, particularly in Gojjam, are *Tella*, *Tej*, and *Areke* [6, 8]. *Borde* is dominated by people living in the Benishangul‐Gumuz region and southern Ethiopia around Hawassa [9]. *Korefe* is predominantly prepared and used in different areas of Gondar. *Tella* is prepared in every part of the country, in both rural and urban areas [8].

*Tella* is similar to other traditional African cereal beers, such as *Burukutu* (West Africa) [10, 11], *Pito* (Nigeria) [10, 12], *Dolo* (Burkina Faso) [13], and *Umqombothi* (South Africa) [14], which rely on mixed microbial consortia, spontaneous fermentation, and household‐level production methods based on local knowledge systems. However, *Tella* uses *gesho* (*Rhamnus prinoides* L.) as a bittering and antimicrobial agent, which has unique phytochemical properties and functions similarly to hops in European beers. *Areke* resembles beverages such as Chinese *baijiu* [15, 16], *Chang′aa* (Kenya) [17], and *Kachasu* (Zambia, Zimbabwe, and Malawi) [18], which are all grain‐based, strong distilled spirits with cultural roots.

Both rural and urban residents can benefit from the use of fermented products [8]. In developing countries such as Ethiopia, local fermented beverages have numerous uses. They may enhance the nutritional value of food, improve its digestibility, prevent harmful organisms from degrading it, and improve its flavor. Furthermore, they are used for medical purposes, leisure activities, wedding ceremonies, religious and nonreligious rituals [8, 19], social events and festivals, during funerals, and as a replacement for food [20]. *Tella* and *Areke* contribute significantly to the local economies of Ethiopia through their roles in local employment, agriculture, tourism, and informal trade [21, 22]. Women are the main producers of *Areke* and *Tella*, and they usually prepare the grains, ferment them, maintain the fermentation process, and distill the fermentation products. The production and sale of *Tella* and *Areke* provide significant sources of income and economic empowerment, especially in rural and peri‐urban areas [23]. Women play an important role in preserving cultural traditions in addition to their economic role, as they frequently offer these beverages during social celebrations and gatherings. They help in the transmission and preservation of social norms, strengthen ties within the community, and preserve the customs around traditional beverage drinking [24, 25]. In recent years, the production of the Ethiopian traditional beverage (*Tella*) has advanced from home‐based settings to small‐scale commercial enterprises that supply bars, hotels, and restaurants in Addis Ababa, Ethiopia [26, 27]. The current development of *Tella* in Addis Ababa, Ethiopia, benefits local economies by generating jobs and income and expanding small enterprises in the hotel industry while maintaining traditional customs [26].

Local alcoholic beverages are made by locals using conventional techniques and ingredients that are easily accessible in the region [28]. Ethiopian conventional techniques and methods of fermented beverage processing are still used with rudimentary equipment made of gourds and wood. Container selection for packaging local beverages by small‐scale manufacturers is frequently limited by accessibility and may consist of earthenware pots and glass bottles. However, reduced shelf life is one of these constraints, and although appropriate for local consumption, it does not help in local market placement and marketing [21]. Inefficient fermentation procedures, low product yields, and inconsistent product quality are some of their drawbacks. In addition, there are safety issues with raw materials associated bacterial pathogens or unsanitary processing methods [28].

*Tella* is a traditional fermented alcoholic beverage produced domestically and sold commercially in Ethiopia. The pH of *Tella* obtained from various locations ranges from 3.87 to 4.67, and the alcohol content ranges from 3.98% to 6.48% (*v*/*v*) [29, 30]. Most people believe this to be a harmless social beverage, and it is often made by women, except in church compounds and monasteries. *Tella* is sold in small houses “Tella bets.” *Tella* is a frequently consumed alcoholic beverage in Ethiopia, which produces approximately 8 million hectoliters of conventionally fermented alcoholic beverages annually [31]. However, brewing in small amounts when there are many consumers results in economic loss because the brewed goods quickly deteriorate and are not immediately consumed or sold [32].

*Areke* or *Katikala* is a distilled fermented alcoholic beverage, colorless, higher alcohol content, indigenous to Ethiopia [33]. It is a traditional homemade fermented alcoholic beverage that is made at home and sold commercially. The alcohol content ranges from 30% to almost 50% (*v*/*v*) [34]. This drink is more expensive than the others and is often thought to be very strong and harmful. *Areke* is produced in rural and semiurban regions, and farmers and people living in this area use it more frequently than city dwellers [35]. Additionally, the majority of *Areke* drinkers in urban areas are either from lower socioeconomic classes or are alcohol‐dependent individuals who cannot afford to purchase industrially made alcoholic beverages [22].

Traditional Ethiopian alcoholic beverages, such as *Areke* and *Tella*, contain varying amounts of phenolics, flavonoids, polyphenols, and tannins, depending on the ingredients and fermentation techniques used [36, 37]. Bioactive compounds are rich in beverages such as *Areke* and *Tella*, especially when made with grains, *gesho* (*R*. *prinoides* L.), and natural herbs. Research indicates that the total phenolic and flavonoid contents of *Tella* are substantially higher than those of *Areke*, which is subjected to distillation and thus retains fewer of these compounds. Tannins are recognized for their antioxidant and astringent qualities and are particularly abundant in *Tella* due to the use of *gesho* and barley [36]. Bioactive substances in traditional beverages are produced during fermentation and offer several biological advantages, such as anti‐inflammatory, anticancer, antidiabetic, antiaging, and antioxidant properties [38, 39]. *Gesho* is a good natural source of polyphenols and flavonoids, which are well recognized for their strong antioxidant activities. The potential health benefits of the antioxidants found in *gesho* include increased bioavailability, prevention of metabolic syndrome–related illnesses, and anticancer effects [40–43].

Locally fermented beverages are a potential source of microbial ecology in Ethiopia. *Tella* and *Areke* have significant potential to develop from conventional home beverages into fully commercialized products. This shift can be achieved through improvements in safety and quality standards, formalization of production procedures, creation of unified branding and marketing strategies, and connection with cultural tourism can all help bring about this shift [33, 44]. Shelf‐life extension, packaging, and standardization of fermentation processes can increase the appeal of products to a broader market. Additionally, they have a unique opportunity to market these local beverages as Ethiopian products with export potential because of their rich cultural heritages. The traditional beverage sector may be scaled up and formalized through government policies that assist small‐scale brewers, offer technical training, facilitate financing, and promote public–private collaborations [21]. Traditional fermentation processes and distillation have the potential to transform domestic arts into contemporary industry necessities through research and technological advancement and/or development. To increase the taste, aroma, and alcohol content of Ethiopian traditional *Tella* and *Areke*, the fermentation process can be scaled up in a way that is appropriate for their localities and affordable for the economy of rural and urban areas by creating an anaerobic environment and using a modified fermenter, starter culture, and distiller [8].

Despite the widespread popularity of *Tella* and *Areke* in Ethiopia, previous review studies have mainly focused on the physicochemical characteristics and preparations [27, 45], as well as their safety and quality [46], and microbiology [47–49]. However, there are limited comprehensive review reports that examine the processing methods in depth and the future commercial potential of *Areke* and *Tella*. Additionally, relatively little attention has been given to the microbial dynamics, metabolic profiling, and functional properties of *Tella* and *Areke*. Therefore, this review is aimed at consolidating studies conducted by numerous researchers on Ethiopian traditional fermented beverages (*Tella* and *Areke*) and providing scientific information on indigenous knowledge, processing methods, microbial dynamics, metabolic profiling, functional properties, and their potential for future commercialization.

## 2. Traditional Alcoholic Beverages

Based on a global alcohol consumption status report, [22] estimates that approximately 30% of all alcohol consumed globally remains unreported. However, this rate is far greater in Africa, Southeast Asia, and the Eastern Mediterranean (56%, 69%, and 31%, respectively). Ethiopia has the highest unrecorded alcohol consumption rate (3.5 L of pure alcohol consumed per capita) compared with other African countries such as Angola (1.6 L), Uganda (1 L), and Nigeria (1 L). Noncommercial (illicit) drinks are described in this review as homemade alcoholic beverages made for domestic use or restricted native trade. It might also comprise a variety of alcohols that are not beverages (produced from pharmaceuticals, automobile items such as benzene, and cosmetics), which is a very common occurrence in certain regions, especially in the lowest socioeconomic groups of problem drinkers [50]. Because these illegal or noncommercial beverages are not recorded, it is challenging to determine their precise alcohol content or the proportion of these local beverages that are manufactured and consumed compared with alcohol that is legally marketed [22].

In Ethiopia, although alcohol plays a significant role in people′s lives, there is a dearth of reliable data about the nation′s alcohol production levels, sold and used, as well as the health issues it causes [29]. Ethiopia is a country rich in ethnic diversity, history, and agricultural genetic variation. Homemade alcoholic beverages are widely consumed by the country′s various ethnic groups as a significant component of local customs surrounding important social events [19]. The authors of [29] demonstrated several indigenous fermented beverages in Ethiopia grouped into high alcoholic beers, such as *Tej*, *Tella*, *Areke* (*Katicala*), and low alcoholic beers, such as *Keribo, Buqri, Shameta, Korefe,* and *Borde*.

Tella is naturally rich in microorganisms that promote health, particularly yeasts and lactic acid bacteria (LAB), which serve as probiotic. Probiotics enhance good nutrition and health by facilitating digestion, reducing intestinal disorders, strengthening the immune system, optimizing gut ecology, and promoting overall health [49, 51]. Several studies have described the probiotic potential of LAB and yeast strains isolated from fermented indigenous beverages. In many in vivo and in vitro studies, several probiotic strains have shown significant cholesterol‐lowering ability, bile salt hydrolase, and antioxidant activities [52, 53]. Three *Saccharomyces cerevisiae* strains (*S. cerevisiae* NP‐7‐5, *S. cerevisiae* WQY‐17, and *S. cerevisiae* var. *boulardii* (nom. inval.) H11) and seven LAB strains (*Pediococcus pentosaceus* TAA01, *Lactobacillus curvatus* TAA04, *Leuconostoc mesenteroides* TDB19, *L*. *curvatus* TDB21, *L*. *mesenteroides* TDB22, *L*. *curvatus* TDM40, and *Lactobacillus plantarum* TDM41) isolated from *Tella* have shown survival under stressor conditions that existed in the host cell. These include tolerance to bile salt, simulated gastrointestinal conditions, surface hydrophobicity, aggregation, antioxidant, and antagonistic activity. The results confirmed that they are probiotics potential [54, 55].

Although traditional fermented beverages are produced in an uncontrolled manner by microbiota, they contain short‐chain fatty acids, vitamins, minerals, antioxidant compounds, bioactive substances, and amino acids [56]. Traditionally fermented beverages provide substantial health benefits in reducing noncommunicable diseases, such as cancer, gastrointestinal disorders, diabetes, immunological disorders, cardiovascular diseases, and allergic reactions [57, 58]. Alcoholic beverages may offer several health advantages when consumed in moderation. However, excessive alcohol consumption has adverse effects on health [23]. Numerous studies have provided evidence that light‐to‐moderate alcohol consumption is associated with higher levels of high‐density lipoprotein cholesterol (HDL‐C), a lower risk of cardiovascular disease, a lower incidence of Type 2 diabetes (T2D), and a decrease in lipid oxidative stress [59–62]. *Tella* contains 0.4 g of protein, 1.98 g of carbohydrates, 0.0 g of fat, 0.0 g of cholesterol, 9.4 mg of calcium, 6.1 mg of magnesium, 9.0 mg of potassium, 2.3 mg of sodium, 0.02 mg of niacin (B3), and 0.093 mg of folate (B9) [63]. *Tella* can provide substantial amounts of vital minerals, such as calcium, magnesium, potassium, and sodium, as well as B vitamins, such as niacin (B3) and folate (B9), particularly in Ethiopia, where malnutrition and micronutrient deficiencies are prevalent [48, 64].

Fermenting microorganisms are essential for improving probiotic activity, safety, digestibility, preservation, nutritional qualities, and antinutritive aspects of fermented beverages [65]. Fermentation improves organoleptic qualities by fermenting microbes that produce unique flavors, textures, and aromas to enhance fermented beverages [4]. The distinct and unique taste of these fermented beverages is mostly due to the cooperative interaction of fermentation metabolites, including lactic acid, ethanol, and other secondary byproducts formed by these microorganisms [4, 34]. LAB synthesizes vitamins (Groups B and K), folate, the amino acid lysine, and micronutrients to increase the nutritional content of traditional beverages [65]. Additionally, fermentation removes unpleasant odors, lowers cooking energy, and destroys antinutritional components such as polyphenols, tannins, and phytic acid [66, 67]. Fermentation prevents spoilage by creating several natural barriers that limit the survival of unwanted microbes [66]. Beneficial fermenting microbes rapidly consume available sugars and nutrients, leaving little for potential spoilage organisms. The carbon dioxide release also aids in limiting the growth of mold and aerobic microorganisms by dislodging oxygen [68].

## 3. Traditional *Tella* and *Areke*

### 3.1. Traditional *Tella*

*Tella* is an Ethiopian traditional beer. It is an opaque alcoholic beverage that ranges in color from light yellow to dark brown (ethanol content 3.98%–6.48% [*v*/*v*], pH [3.87–4.67]), and is indigenous to Ethiopia [29, 30]. A variety of grains are used to make this malt beverage, such as sorghum (*Sorghum bicolor* L.), teff (*Eragrostis tef* L.), millet (*Eleusine coracana* L.), barley (*Hordeum vulgare* L.), wheat (*Triticum sativum* L.), and maize (*Zea mays* L.) are a few examples [8, 45, 69]. *Tella* brewing techniques in Ethiopia vary slightly between areas and ethnic groupings, depending on the brewers′ economic circumstances and traditions [63].

Filter *Tella* is another beverage, which is made in a similar way as regular *Tella*, but it is more concentrated (5%–6%) [33]. Filtered *Tella* and normal *Tella* are very similar in terms of their ingredients, preparation techniques, and cultural importance [70]. Both are fermented with *enkuro* (toasted flour), *kita* (flatbread), and *gesho* (*R*. *prinoides* L.) using barley, wheat, maize, and other grains [69, 71]. However, normal *Tella* is unfiltered and has an earthy flavor, thicker texture, and cloudy appearance because of suspended solids, such as *enkuro* and other residues. In contrast, filtered *Tella* is subjected to further sifting and settling to remove suspended particulates and produce a smoother, clearer, and milder beverage. Normal *Tella* tends to be lower in alcohol and more variable, especially when taken fresh, but filtered *Tella* has a higher and more consistent alcohol level due to its longer fermentation and filtration process [30, 33, 69].

*Tella* is the most widely consumed drink in the Amhara, Tigray, and Oromia regions of Ethiopia. The raw materials and methods used to produce *Tella* differ depending on the ethnic group, economic status, and customs. Depending on ingredients used to make *Tella*, *Tella* is known by a variety of names: Amhara′s *Tella*, Oromo′s *Tella*, and Gurage′s *Tella* [27, 48, 72]. Amhara *Tella* is distinguished by its *gesho* (*R*. *prinoides* L.) composition and its comparatively high ethanol level. Many types of spices are used to delicately aromatize Gurage *Tella*. Oromo *Tella* is distinguished from Amhara or Gurage *Tella* by its distinct preparation techniques, ingredients, and flavor attributes [48]. Oromo *Tella* differs from other Ethiopian *Tella* in that it does not contain *gesho*, a bitter and antimicrobial ingredient frequently used in Amhara and Gurage recipes [73, 74].

### 3.2. Cleaning Agents Used in *Tella and Areke* Preparation

*Grawa* (*Vernonia amygdalina* L.) and *weira* (*Olea europaea L. cuspidate* L.) are used to clean fermentation containers prior to the *Tella* and *Areke* fermentation process [32]. Fermenting vessels (*ensira* or *gan*) are cleaned with *grawa* leaves, and *weira* splinters are used for smoking fermentation vessels. *Grawa* and water are used to repeatedly cleanse *ensira* or *gan*, and after that, it is smoked for roughly 10 min using dry wood from *weira*. Smoking the traditional clay pot (*insera*) gives *Tella* a distinct earthy aroma, flavor, microbiological stability, and smokiness that are crucial to its sensory profile and cultural significance [69].

### 3.3. Raw Materials Used in *Tella* and *Areke* Production

Barley or wheat‐germinated grains (*bikil*), either made at home or purchased from the neighborhood market. The grains used to make *bikil* are soaked in water, sandwiched between fresh banana or castor leaves, and allowed to germinate for 3 days. After germination, barley grains are sun‐dried and ground to produce malt flour [6]. Malted grains (*bikil*) are an essential component of *Tella* or *Areke* fermentation preparation that provides fermentable sugars and enzymes, such as amylases, which are activated during the malting process and breakdown to starch [75]. Beyond its enzymatic role, *bikil* also introduces a variety of wild yeasts and bacteria due to its unsterilized nature [32]. The use of traditional fresh leaves to cover malt during malting has a scientific basis, as it creates a regulated microenvironment for germination [76]. Fresh leaves assist grains in maintaining adequate moisture for consistent sprouting by slowing evaporation. Leaves regulate temperature fluctuations as organic insulators and promote consistent germination. Additionally, they promote balanced aeration and minimize contamination by limiting exposure to dust, insects, and microbial spoilage [76, 77].

The two primary sources of carbohydrates used in the preparation of *Tella* and *Areke* are *kitta* and *enkuro.* To prepare *kita* (a 5–10‐mm thick, thin, pancake‐like bread), the first step in making *kitta* is mixing water with flour from barley, wheat, and maize to form a dough. The dough is then formed into round, flat pieces and baked on a *mitad* (traditional clay griddle). *Kitta* is allowed to cool and then broken up into smaller pieces after baking to increase the *kitta* surface area and appropriate it for fermentation. This crushed *kitta* is an essential fermentable sugar source for the production of *Tella* [8, 32, 69, 78]. To prepare *Enkuro* (toasted flour), first, the maize is soaked in water for about 3 days, and after that, it is dried, roasted, and ground to make “enkuro,” a dark maize flour that is the primary component that gives *Tella* its color. The roasted flour is ground into a powder and placed on a hot *bret mitad* (griddle composed of a thick, flat piece of iron) after being sprayed with water and steamed to make *enkuro*. The brewer controls the amount of heat treatment between 70°C and 100°C [8, 48].

*Gesho* (*R*. *prinoides* L.) is basic ingredient used for producing traditional alcoholic beverages in Ethiopia, particularly *Tella*, *Areke,* and *Tej*, which serve as a bittering and flavoring agent [33]. *Gesho* is a local hop that is accessible from the local market. *Gesho* leaves and stems are the necessary ingredients used to make *Tella* and *Areke.* The leaves and stems of “gesho” (*R*. *prinoides* L.) are sun‐dried and ground [8]. *Tella* bitterness is proportional to the quantity of *gesho* added during the brewing process [33]. Similar to hops, *gesho* leaves are used in the traditional brewing process to provide a bitter taste and antibacterial properties to the final product. *Gesho* possesses several characteristics, such as an antibiotic effect, citrus, herbal scents, and flavors. These properties contribute to its traditional use in various alcoholic beverages, which are desirable to many brewers [79]. The chemical that gives it its bitterness is called *β*‐sorigenin‐8‐O‐*β*‐D‐glucoside (geshoidin) a naphthalenic molecule [41].

### 3.4. Steps in *Tella* Preparation

**Figure 1:**
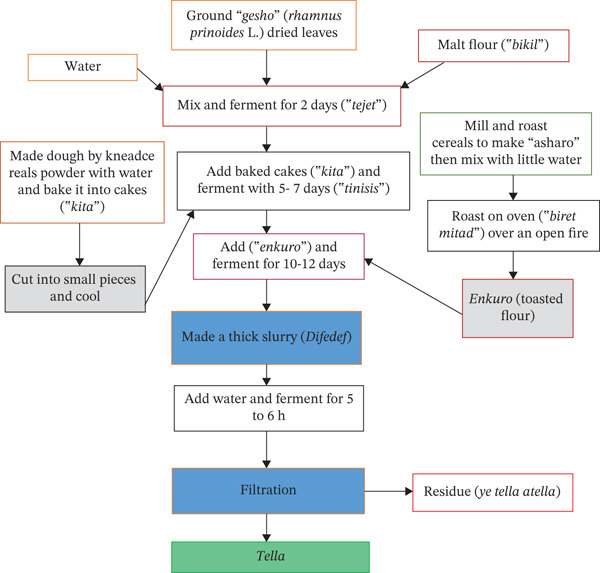
Flow diagram of the traditional *Tella* production method [32, 73].

*Tella*′s traditional fermentation process consists of three main stages, each distinguished by the addition of ingredients at various periods. Although local variations in the process are slight, the fundamental steps are the same throughout the country. The method of production resembles that of beer, and the presence of *gesho* serves a similar purpose as hops in beer [33, 80]. However, the fermentation process does not involve a yeast inoculation step; instead, it uses the natural microorganisms found on cereals, utensils, and in the environment [7, 32]. The three fundamental steps in the *Tella* production procedure involve making “tenses,” “difdif,” and “Tella” [32, 33, 73] (Figure 1).

#### 3.4.1. Phase I

Leaves and stems of *gesho* powder (*R*. *prinoides* L.) (1 kg), *bikil* (malt) (0.5 kg), and water (12 L) are added and properly mixed in traditional fermenters. Subsequently, the ingredients in the fermenter were left to ferment for 3 days at room temperature. Then, a clean, smoking traditional bioreactor called an “insera” is used to mix “gesho” powder and “bikil” flour with a sufficient amount of water. *Tejet* is produced by fermenting this mixture for 2 days. This fermenting substance is frequently called “tejet.” At the end of the fourth day, *tejet* fermentation is completed, *tejet* in fermenters, water (15 L), powdered malt, powdered leaves of *gesho*, and 5 kg of *kitta* (unleavened bread) are added, and for an additional day, the content in fermenters is left to ferment. This stage is referred to as “tinisis” and is the starter culture for *Tella* fermentation.

#### 3.4.2. Phase II

Maize (*Z*. *mays* L.) is soaked in water for approximately 3 days, after which it is dried, roasted, and ground to make *enkuro* (toasted flour) on a hot *bret mitad* (griddle composed of a thick, flat piece of iron). *Enkuro* (10 kg) is then added to the previously produced “tenses” and anaerobic fermentation for 10–20 days. After this fermentation period, a thick slurry, known locally as “difdif” is formed. In the fermenter, *difidif* was fermented for another day.

#### 3.4.3. Phase III

Water was added after the fermentation of “difdif” was completed in the fermenters, 12 L of water was added, and the mixture was properly mixed. Water was added in sufficient amounts to fill the fermenters. The container was completely filled with water in the final stage, and the slurry was well mixed. This stage is a dilution stage that helps in the proper completion of the fermentation process. The filled fermenters were then covered with plastic material to create an anaerobic environment and left for 1–2 days. *Tella* is consumed either unfiltered or filtered.

### 3.5. Traditional *Areke*

*Areke* is a clear, colorless, higher alcohol distilled beverage, an indigenous Ethiopian distilled spirit [33]. *Areke* is produced by distilling from the fermentation mash made out of cereals, water, and ground *gesho* (*R*. *prinoides* L.). A variety of grains are used to make *Areke* beverage, such as barley (*H*. *vulgare* L.), wheat (*T*. *sativum* L.), maize (*Z*. *mays* L.), millet (*E*. *coracana* L.), sorghum (*S*. *bicolor* L.), teff (*E*. *tef* L.), and others [6, 69]. Local *Tella* can be distilled to make *Areke*. *Areke* is classified into two traditional categories: *Terra-Areke and dagim-Areke*. In Amharic, the phrase “terra” means “ordinary,” whereas “dagim” means “second time,” indicating that it has been distilled twice. *Terra-Areke* has a reported alcohol level of 34.09% (*v*/*v*), with a range of 30.20%–39.90% (*v*/*v*) [30, 81]. *Dagim-Areke* is stronger than *terra-Areke*, which is made similarly to *terra-Areke*, except that the distillation process is given less time, or *terra-Areke* is redistilled in three volumes to obtain approximately one *dagim-Areke* volume [81]. The alcohol content of the redistilled *Areke* then increases. *Dagim Areke* usually has an alcohol concentration of about 45% (*v*/*v*) [30].

Big pot (*metensesha*) is the local alcohol distiller uses a big pot as a fermenter. Clay pot (*madiga*) is a small clay pot used to boil the fermented mash to releasing vapor. Cloth is used to seal the *madiga* to prevent vapor loss. Bamboo tube is made of hollow plant steam and used as a pipe to transport vapor from the clay pot (boiler) to the collector. *Koda* is used as a vapor collector that comes through a bamboo tube and is made from aluminum or metal. *Tofaa* is made of clay used for containing cooling water in which the metal bottle or *koda* that contains vapor for changing the vapor into liquid [82].

The final product of alcohol content, flavor, and purity is greatly affected by the intensity of heating used during *Areke* distillation. Steady heating is advised to increase the alcohol content and improve purity. The final product of alcohol content, flavor, and purity is greatly affected by the heating intensity used during *Areke* distillation. Steady heating is advised to increase the alcohol content and improve purity. In contrast, high‐intensity heating results in inadequate separation, reduced alcohol content, and an unpleasant flavor [82–84].

### 3.6. Steps of *Areke* Preparation

**Figure 2:**
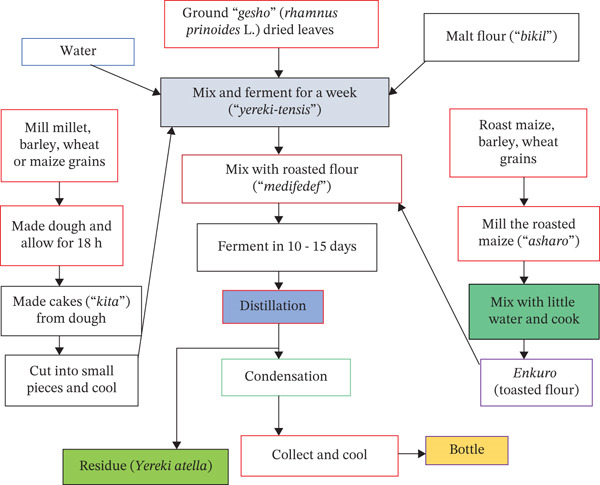
Flow sheet of the traditional *Areke* production method [84, 85].

*Areke* is distilled from fermentation products made in a manner similar to *Tella*, except that the fermentation mass is more concentrated in this instance [34]. *Yereke-tensis*, *medifedef,* and *Areke* are the three basic processes in the *Areke* production process [6, 84, 85] (Figure 2). At the initial stage, *yereki-tensis* is made by mixing powdered *bikil* and *gesho* leaves in a 1:2 ratio with water to produce a free‐flowing combination that is then left to ferment for a week [81]. To create *medifedef*, cereals (sorghum, teff, millet, barley, wheat, and maize) were soaked, dried, roasted, and ground to make *kita* (thin pancake‐like bread) and *enkuro* (toasted flour). Enkuro was prepared by steaming roasted maize flour on *a bret mitad* at 70°C–100°C, whereas *kita* was cooked on a *mitad*. Finally, 20 kg of *enkuro* and 5 kg of *kita* were added to *yereki-tensis* and fermented anaerobically for 10–15 days. However, this depends on the season and ambient temperature of the environment. When the temperature of the environment becomes hot, it can be completed within 10 days. Traditionally, *Areke* producers decide on the completion of fermentation by looking at the fermented mash sludge. If the sludge at the top of the fermented mash is changed into liquid, it is said to be the end of fermentation [32]. The fermented mash was boiled for *Areke* distillation after fermentation was complete.

### 3.7. Distillation Method of Traditional *Areke*

**Figure 3:**
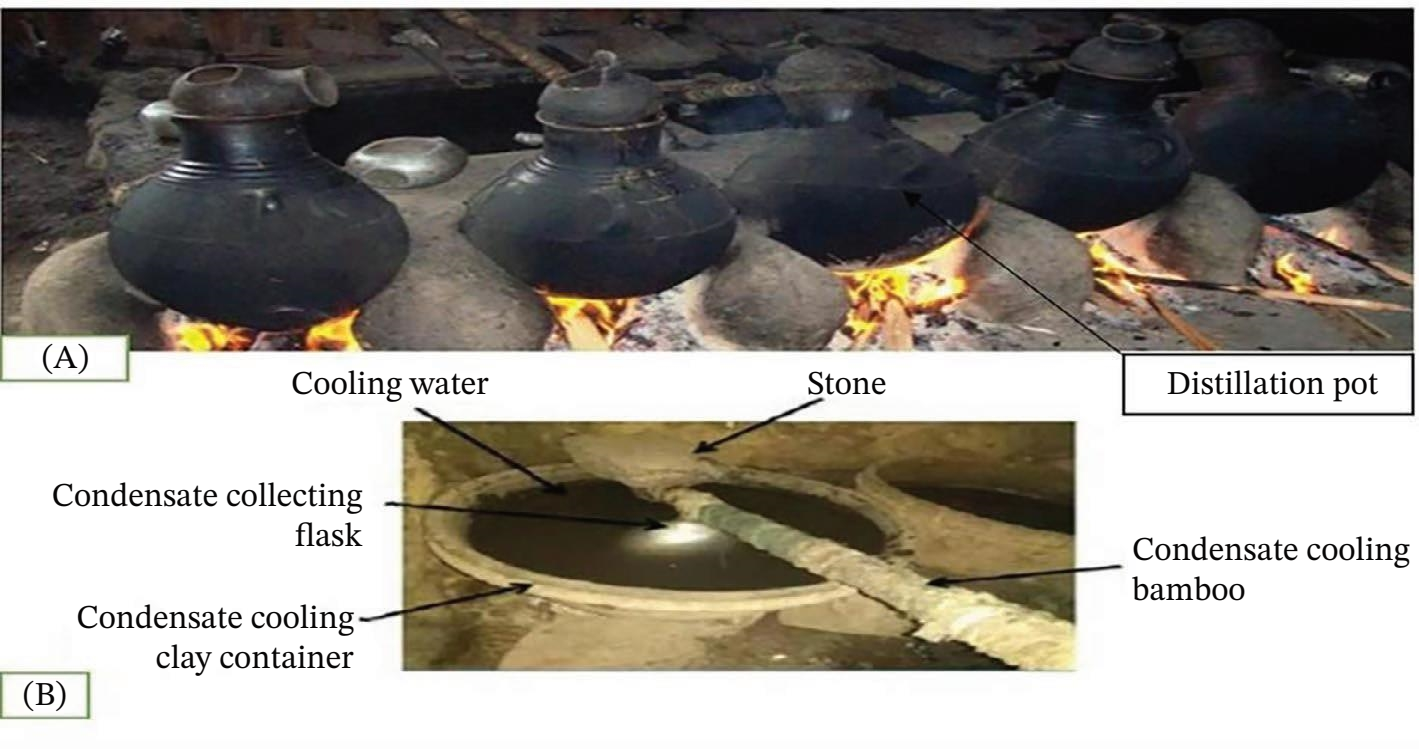
Traditional *Areke* distillation: (A) Traditional distillation *Areke* with local stove, and (B) process of traditional condensation [86, 87].

The method of *Areke* distillation was described by [82, 86–88] (Figure 3). First, the clay pot (*madiga*) is placed on three stones. The upper part of the clay pot is then covered with a small bowl‐shaped clay pot. Firing is performed with firewood in the middle of the three stones. When the clay pot becomes hotter, the fermented mash is transferred from the fermenter to the boiler (clay pot) by leaving some space at the upper surface of the clay pot and boiling for a few hours. From firing until the mash starts to boil, the bamboo tube (*shembeko*) is not attached to the clay pot and *toffa* and the hole for attaching the tube is not sealed. When the fermented mash starts boiling, the bamboo tube (*shembeko*) is adjusted and attached to the head of the clay pot and sealed using cloth and mud to prevent vapor losses from the clay pot. As the process continues, alcohol vapor evaporates and condenses in a bamboo tube. The adjustment of a bamboo tube (*shembeko*) is slanted down from the bowl‐shaped head cover to the *koda.* This adjustment helps as the condensed vapor drops into the *koda* by gravitational force. *Koda* is placed in a *toffa* or half clay pot filled with cold water. Cold water was used to cool the collected product. When it becomes hot, it is changed by cold water. The collection of drops and cooling occurred simultaneously at the same location inside the *toffa*. When the *koda* becomes full, distillation is stopped, and the *Areke* is transferred to another bottle. The *koda* is adjusted again, and the process continues. The collected product during this time is weak in its alcohol content and is called *bulus*. The residue from the clay pot is cooled and used as feed for cattle. Recooling the water in the *toffa* is essential for maintaining effective condensation, increasing alcohol yield, improving purity and flavor, and ensuring safety and consistency in *Areke* distillation [82].

The lengthy process of *Areke* distillation takes more than 12 h. The distillation process takes a long time because there is no improvement in the system. Processing a single distillation batch takes 2.5 h. The longer distillation time is due to the fermented mash, which is a mixture of ethanol, water, and solid residue, and it is heated directly. Unlike the majority of local cooking practices, *Areke* distillation necessitates cautious fire management, and the cook must be present in the kitchen once the procedure begins to monitor any changes that can affect the product′s quality (the amount of alcohol and the product′s flavor). Controlling the stove′s fuel consumption is necessary to prevent heating the distillation above the alcohol′s boiling point or overheating the pot, which causes the contents to adhere to the bottom. Any steam that might escape through the condensation tubes at the two ends where they are connected to the pot, and maintaining the temperature gradient along the condensation tube requires checking the collecting flask and keeping the water in the cooling tube at a low temperature. During this distillation period, the amount of firewood consumed and the amount of *Areke* distilled depend on the condenser, energy‐saving stoves, and distillation equipment, which results from the average amount of time spent using each stove during the distillation process [86, 87].

They use an open fire system in which much of the heat is wasted to the environment. This forces them to use much more firewood. Thus, the energy utilization system is not economical. The transportation and condensation of vapor are carried out by a bamboo tube made of a hollow plant stem. There is no better cooling system for the tube to cool and condense high‐heat vapor quickly to reduce losses [85]. The heat supplied in the boiling stage significantly affects the quality of the final distilled *Areke, including its* flavor, aroma, color, and alcoholic strength. There is no method to control the product quality, alcohol content, and the purity of alcohol in *Areke* while producing it traditionally at home. If overheating occurs during the boiling of the mash, the residue burns at the bottom of the *madiga* (clay pot for distillation), and hence, the flavor and color are affected [86]. Batch uniformity, high alcohol content, safe and clean flavor, and quality and safety can only be achieved through *Areke* distillation when the right amount of heat is maintained. Inefficient distillation, where the heat is set too low, results in long distillation times and a low alcohol yield. However, excessive heat can lead to overheating, which can release contaminants and pose a risk to public safety [84, 86].

## 4. Microbiological, Physicochemical, and Functional Properties of *Tella* and *Areke*

*Tella* and *Areke* traditional alcoholic beverages are produced through spontaneous fermentation by complex and dynamic microbial consortia. LAB, yeasts, and, in the early phases, different aerobic mesophilic bacteria and Enterobacteriaceae dominate the fermentation microbiota [32, 48, 54, 63, 70]. Microorganism succession influences the safety, flavor, nutritional quality, and functional qualities of traditional alcoholic beverages [63]. LAB are the most dominant bacteria found in *Tella* and *Areke* fermentations. *Lactobacillus* species, such as *Lactobacillus pastorianum and Lactobacillus postanumi* are frequently described [54, 63].

LAB create an acidic environment through lactic acid production, and prevent other unwanted microorganisms by reducing the pH to less than 4.5 [89]. Bacteriocins, hydrogen peroxide, diacetyl, and organic acids are among the antimicrobial metabolites that LAB produce in addition to lactic acid. These metabolites aid in the development of flavors and the stability of products [90]. Glycolysis, lipolysis, and proteolysis are among the metabolic pathways that LAB primarily use to shape the flavor, texture, and nutritional value of the final products [91]. LAB improves the nutritional value of beverages by synthesizing micronutrients, folate, the amino acid lysine, and vitamins (Groups B and K) [92, 93]. Yeast converts fermentable sugars into ethanol and carbon dioxide through alcoholic fermentation [94]. The most frequently isolated species of *Tella* and *Areke* are *Saccharomyces* species (*S. cerevisiae* and *Saccharomyces carlsbergensis*) [63].

Based on metagenomic 16S rRNA sequences, *Tella* samples contain lower bacterial richness and diversity than other Ethiopian traditional fermented beverages, such as *Bord*e, *Cheka*, *Shamita,* and *Kinito*. The most abundant genus was *Lactobacillus* (77.22% of the total community), followed by other LAB (*Lacticaseibacillus* [4.29%], *Companilactobacillus* [2.65%], *Paucillactobacillus vaccinostercus* [1.21%], and *Lactiplantibacillus* [0.95%]), Enterobacteriaceae (1.74%), and the remaining taxa (11.87%) [95].

According to a report by Wubie [96], the physical characteristics of *Tella*, including electrical conductivity, salinity, total dissolved solids (TDS), total suspended solids (TSS), and total solids (TS), are 2359 *μ*S/cm, 1.20%, 1180 mg/L, 540 mg/L, and 1721 mg/L, respectively. *Tella* chemical composition includes 2.86‐g/L fixed acidity, 30.6‐g/L volatile acidity, and 33.5‐g/L total acidity, which have contributes to its sour taste, preservation stability, and overall quality of *Tella*. The physical characteristics of *Areke* include a mean electrical conductivity of 18.89 *μ*S/cm, specific gravity of 0.94, degree of Brix of 15.77 ± 2.36, and TDS of 9.42 *μ*S/cm. The chemical composition of *Areke* includes methyl alcohol, ethyl alcohol, aldehydes, and esters at concentrations of 0.49% (*v*/*v*), 34.9 (*v*/*v*), 0.29 g/L, and 0.26 g/L, respectively. In addition, the mean total acidity is 0.90 g/L, whereas the mean volatile and fixed acidities are 0.67 and 0.11 g/L, respectively [97]. The mean concentrations of total phenolics, flavonoids, and tannins, and antioxidant capacity in various *Areke* samples are reported as 0.28 *μ*g mL−1 GAE, 0.077 *μ*g mL−1 CE, 0.04 *μ*g mL−1 GAE, and 99.5 *μ*g mL−1 AAE, respectively. In comparison, the average levels of total phenolics, flavonoids, tannins, and antioxidant capacity in various *Tella* samples are 13.73 *μ*g mL−1 GAE, 5.46 *μ*g mL−1 CE, 1.98 *μ*g mL−1 GAE, and 116.69 *μ*g mL−1 AAE, respectively [36].

Aroma compounds play an important role in the sensory qualities of traditional fermented beverages [98]. The aroma compounds in traditional alcoholic beverages are a mixture of volatile and nonvolatile compounds that are produced during fermentation. These compounds include alcohols, esters, aldehydes, ketones, organic acids, and phenolic compounds, which are responsible for the aroma and flavor of the beverage [99, 100]. The primary organic acids produced by the LAB strain from *Tella* include lactic acid (1.49 g/L), acetic acid (0.16 g/L), citric acid (0.71 g/L), malic acid (1.11 g/L), and succinic acid (0.74 g/L) [95, 101]. Yeast and LAB are primarily responsible for the production of aroma compounds through the metabolism of sugars, amino acids, and other nutrients [102].

According to Teshome et al. [103], the aroma characteristics of various *Areke* samples are characterized by fruity and floral esters, with the presence of ethyl esters such as hexanoate ethyl, octanoate ethyl, dodecanoate ethyl, palmitate ethyl, and stearate ethyl. These esters are generally known to be responsible for pleasant aroma characteristics, which are described as fruity, sweet, pineapple‐like, banana‐like, waxy, and creamy aromas. Isoamyl acetate, which was found in some *Areke* samples, is responsible for a sweet banana flavor, whereas 2‐phenylethanol is responsible for a rose‐like floral character [103, 104]. The presence of these fermentation‐derived esters indicates that the various *Areke* samples have a strong fruity floral base aroma, which is typical of distilled spirits produced by yeast fermentation [103, 105].

A high concentration of methanol has been detected in the *Areke* distilled beverage samples, especially wheat *arefa Areke* (7649 mg/L), *yemar Areke* (7518 mg/L), and *dagim Areke* (7504 mg/L), but not detectable in different samples of *Tella* [34]. The elevated concentration of methanol is mainly dependent on the distillation process, where methanol is concentrated because of its volatile nature [97].

## 5. Major Constraints on Fermented Beverages

Hygiene and quality control are crucial for the home‐made beverages successful marketing [106]. Quality standards must be adhered to at the production site. The critical control point for hazard analysis and guidelines for good manufacturing practices should be considered when producing products in a hygienic manner [21]. The products to be commercialized needs a consistent and optimized method that can be documented for future use. Global adoption of these standardized procedures can help local products gain international recognition.

Standardization and intellectual property of the product and reproducing homemade recipes on an industrial scale are the largest obstacles that processed traditional beverage firms are currently facing [107]. Despite their many benefits, traditional beverages have several drawbacks that prevent them from being commercially marketed. These include poor hygiene and quality control by producers and handlers, absence of a standardization protocol that is acceptable for a larger population, insufficient logistics for expanding to large‐scale production, insufficient understanding of appropriate packaging materials and transportation systems, insufficient institutional support systems, absence of trademarks and branding on produced goods, absence of market networking, insufficient accessibility and availability of processing equipment and technical know‐how on handling, lack of training to help entrepreneurs develop their skills, and lack of managerial and marketing skills for entrepreneurship development [21, 44].

Poor sanitation and inappropriate posthandling fermentation are somewhat linked to the existing low technical fermentation technique (inadequately dried, not chilled, or pasteurized), which can render products with a short shelf life and a high potential for contamination. Likewise, many small‐scale producers package their fermented products in locally available materials, which, although acceptable for local consumption, are often unfavorable for prolonging shelf life or being attractive at the point of sale. However, in addition to the technical constraints facing small‐scale processors, several institutional limitations exist, including the inadequacy of public policies to promote and support small‐scale fermentation processing; poor rural communities tend to have inadequate infrastructure, and access to external inputs and professional help is sometimes constrained [106]. Any displacement of local fermentation in developing countries, with technologies developed in more affluent countries, could lead to centralized manufacturing, issues with distribution, less participation of locals in fermentation processing, less employment in certain regions, less nutrient‐dense substitutes for basic materials, replacement of traditional arts, loss of unique local know‐how, and dependency on equipment imports and resources, and might not otherwise fully satisfy regional needs like conventional fermented products [21].

The production of *Tella* is still based on spontaneous fermentation techniques, which lead to significant variations in its consistency, safety, and quality [48]. The composition of microorganisms is uncertain and may contribute to the development of harmful substances, including methanol, acrylamide, and other volatile congeners, as well as pathogenic and spoilage microbes [50]. The limited ability of traditional Ethiopian alcoholic fermentation vessels to control temperature, pH, and aeration leads to inconsistent product quality [71]. Variations in the raw materials, fermentation vessels, microbial inoculation processes, and various regional adjuncts are used in different regions of Ethiopia for traditional *Tella* preparation methods. This disparity usually results in significant differences in producer quality, flavor, and alcohol content, which reduces customer confidence and commercial viability [46, 71].

The shelf life of *Tella* is very short, ranging from 5 to 7 days. This prevents it from reaching larger markets and is only distributed locally. *Tella* shelf life cannot be extended without appropriate preservation and packaging technology, which reduces the producers ability to generate income from the product [63]. Traditional *Tella* producers operate at the household level and have little access to funding, modern fermentation equipment, long shelf life, or technological expertise. Additionally, there is often a lack of government support, research, weak regulatory frameworks, and investment to transform traditional beverages into commercially viable products [44].

## 6. Opportunities and Future Prospects for Fermented Beverages

There are opportunities to scale up and increase the effectiveness of certain fermentation processes. However, every action taken to support livelihood activities needs to be carefully planned, culturally sensitive, and consider the available assets and resources. Working in partnerships or joint ventures with local agroindustries and being well‐organized are essential for successful operations, and universities or wholesalers can assist small‐scale producers in lowering their risk and vulnerability [44]. Traditional fermented beverages provide substantial financial opportunities by creating jobs and revenue for the communities nearby [21, 22]. Home brewers can expand their products into small‐scale production at bars, hotels, and local restaurants. Traditional beverages can be standardized and packaged to satisfy modern market demands by increasing consumer interest and improving their value chain from the supply of raw materials to retail. These advancements enhance local and national economies and improve livelihoods [21, 44]. By branding them as unique traditional products, beverages may be marketed as an important cultural component of heritage and identity, opening up opportunities for both tourism and cultural promotion [106].

Reducing batch‐to‐batch variation and improving safety can be achieved by standardizing fermentation using predefined starter cultures and building microbial repositories [8, 108]. The shelf life of *Tella* is extended by up to 14 days with vacuum filtration and pasteurization, which are necessary for modernizing traditional beverage production techniques [63]. *Tella* storage under cold storage is also used to improve the stability of its shelf and preserve its quality [109]. Standardized processes and routine testing of methanol, alcohol concentration, and microbiological load of traditional beverages are used to reduce regulatory and safety concerns [21].

Research and innovation are necessary to establish a connection between these traditional beverages and international markets [110]. The consistency, shelf life, and health‐promoting properties, including probiotics, have been enhanced by developments in microbial profiling, fermentation optimization, and starter culture production [111]. Modern scientific research and innovations by integrating traditional knowledge can market these beverages as premium, culturally diverse substitutes, successfully connecting traditional products with current worldwide consumption trends [110].

Developing new business models and building consumer trust by promoting traditional beverages as culturally authentic and scientifically validated functional products [58, 112]. This strategy promotes traditional beverages through branding and storytelling to emphasize their probiotic advantages, cultural significance, and heritage, while using digital barcodes to offer openness by disclosing fermentation procedures, safety measures, and microbial composition [112, 113]. Continuous consumer education programs build a solid foundation for market confidence and trust by increasing awareness of natural probiotics, gut health, and the inherent value of traditional beverages [112].

Continuous innovation through omics‐driven research is essential for identifying metabolic pathways, functional capabilities, and microbial diversity in traditional fermented beverages [110, 114]. This approach begins with microbial bioprospecting to identify various native strains, followed by whole‐genome sequencing to discover advantageous genes associated with probiotic characteristics, ethanol production, enzyme synthesis, detoxifying capabilities, and safety markers [110, 115]. The application of innovation and gaining an advantage in the market, manufacturers use omics‐driven insights to investigate new product formulations, flavor profiles, and value‐added functional beverages, in addition to optimizing and stabilizing fermentation processes [114, 115]. The integration of omics technology in traditional fermented beverages can facilitate the use of promising microbial strains in the industry, optimize fermentation processes, improve product safety and consistency, and help in the standardization and commercialization of traditional alcoholic beverages [115].

Commercializing Ethiopian traditional alcoholic beverages, such as *Tella* and *Areke,* can bring economic benefits and improve food and nutrition security. This scale‐up strategic approach can be achieved by defining starter cultures to increase consistency, optimizing fermentation processes, investing in suitable equipment, establishing a regulatory pathway, emphasizing sustainability, and educating consumers about the economic value of these traditional beverages [8, 21]. Private sector investment in specialized fermentation equipment, such as bioreactor design and process automation, is a significant factor in the shift from traditional beverage production to standardized large‐scale commercial operations [116]. Temperature, aeration, and microbial activity are uncontrolled in traditional alcoholic fermentation, which occurs in natural vessels, such as clay pots, gourds, or wooden containers. This often leads to differences in safety, quality, and taste [71]. Designing a bioreactor addresses these problems by enabling the optimization of the fermentation process, thereby reducing expenses and production time by providing optimal conditions for microbial growth and the synthesis of the desired microbial fermentation products [117].

Universities must provide comprehensive training courses, workshops, and extension services to address the needs of local producers and business owners engaged in the *Areke* and *Tella* value chains [118]. Universities are also essential for technical innovation, bringing modern equipment to local producers, and encouraging developments in areas such as quality assurance, distribution, and packaging [119, 120]. They offer extension services and training that empower local producers and business owners while expanding knowledge in areas such as brewing methods, distillation apparatus, and fermentation science [121].

## 7. Conclusions

The production of local traditional beverages is related to the region′s abundant native knowledge system, which is intrinsically linked to its organizational, social, cultural, and environmental contexts. Traditional fermented beverages have numerous uses in developing countries, such as Ethiopia. It can enhance the flavor of beverages, improve the ability of food to be digested, prevent harmful organisms from degrading food, and improve its nutritional content. Further, it is utilized for therapeutic purposes, recreational purposes, in marriages, naming, rain‐making ceremonies, disputes, in religious and nonreligious ceremonies at festivals and social events, and at funeral services. Traditional *Tella* and *Areke* are the most popular alcoholic beverages in Ethiopia. They are produced on a small scale and sold by local alcohol vendors from their homes. Traditional *Tella and Areke* fermentation are a spontaneous and uncontrollable process that involves the natural growth of microorganisms. Raw materials, utensil, and the surrounding environment are sources of microorganisms used in the fermentation process. The equipment, methods, ratios, and ingredients used to prepare these beverages differ depending on the location. These conventional alcoholic beverages show unpredictability in quality between and within productions and have a limited shelf life. Ethiopian traditional alcoholic beverages, such as *Tella* and *Areke,* have tremendous commercial, cultural, and scientific potential, as they are modernized and standardized. Modern fermentation technologies, such as microbe profiling, starter cultures, and omics‐driven innovation, can be combined with traditional knowledge to improve product safety, consistency, shelf life, and preserve cultural authenticity. Additionally, market confidence and international recognition can be increased by storytelling, branding, consumer education, and positioning these traditional beverages as beneficial, culturally rich products with probiotic and health‐promoting properties. *Tella* and *Areke* can be marketed and standardized using starter cultures, enhancing fermentation methods, investing in the appropriate equipment, creating a regulatory pathway, and informing consumers about the benefits of these traditional beverages.

**Nomenclature**
CSA: Central Statistics Authority
EPHA: Ethiopia Public Health Association
FAO: Food and Agricultural Organization
ICAP: International Center for Alcohol Policies
WHO: World Health Organization

## Funding

This study was supported by the Debre Markos University (10.13039/501100021567).

## Conflicts of Interest

The author declares no conflicts of interest.

## Data Availability

Data sharing is not applicable to this article as no datasets were generated or analyzed during the current study.
